# Measurement-Based Modelling of Material Moisture and Particle Classification for Control of Copper Ore Dry Grinding Process

**DOI:** 10.3390/s21020667

**Published:** 2021-01-19

**Authors:** Oliwia Krauze, Dariusz Buchczik, Sebastian Budzan

**Affiliations:** Department of Measurements and Control, Silesian University of Technology, Akademicka Street 16, 44-100 Gliwice, Poland; dariusz.buchczik@polsl.pl (D.B.); sebastian.budzan@polsl.pl (S.B.)

**Keywords:** moisture, moisture modelling, pneumatic classification of particles, particle size, grinding, electromagnetic mill, bulk materials, copper ore processing

## Abstract

Moisture of bulk material has a significant impact on energetic efficiency of dry grinding, resultant particle size distribution and particle shape, and conditions of powder transport. As a consequence, moisture needs to be measured or estimated (modelled) in many points. This research investigates mutual relations between material moisture and particle classification process in a grinding installation. The experimental setup involves an inertial-impingement classifier and cyclone being part of dry grinding circuit with electromagnetic mill and recycle of coarse particles. The tested granular material is copper ore of particle size 0–1.25 mm and relative moisture content 0.5–5%, fed to the installation at various rates. Higher moisture of input material is found to change the operation of the classifier. Computed correlation coefficients show increased content of fine particles in lower product of classification. Additionally, drying of lower and upper classification products with respect to moisture of input material is modelled. Straight line models with and without saturation are estimated with recursive least squares method accounting for measurement errors in both predictor and response variables. These simple models are intended for use in automatic control system of the grinding installation.

## 1. Introduction

Grinding is one of the most important technological processes used in many branches of industry. For example, in the construction industry, grinding is used to crush components of building materials; in metallurgy–for metal ores; in the chemical and pharmaceutical industries–for substrates and reaction products; in the food industry–for food ingredients and semi-finished products; in power plants–for coal in coal pulverizers [1].

Moisture of bulk material plays a very important role in the grinding process. It has a significant impact on energetic efficiency of grinding, resultant particle size distribution and particle shape [2,3,4,5]. Too dry powders pose the risk of explosion [6,7,8], whereas too wet–clog transport pipes and other installation elements [9]. In addition, usability of the grinding product (as a final product or as input for following technological processes) often depends on contained moisture, e.g., because of its effect on powder flowability [10], storage methods, and durability (shelf life) [9]. Summarizing, material moisture has many effects on grinding process and on behaviour of powders. Thus, moisture content needs monitoring and control on many stages: in input material and final product, and in intermediate material streams as well [9].

The influence of moisture of bulk materials on the process of their grinding and separation in pneumatic transport has been raised in many publications. Authors of [2] focused on the overall and the specific comminution efficiency of a circuit consisting of a high-pressure-grinding-rolls (HPGRs) unit followed by a batch ball mill as a function of the moisture level in the HPGR input material. The authors noticed that dry material showed the lowest particle size reduction ratios irrespective of the specific grinding force level. A comprehensive study of the effect of moisture material on the grinding process in the food industry was described in [3]. Authors presented in detail the influence of moisture content on the grinding characteristics, grinding methods for food materials, including dry, semi-dry, and wet grinding. The effect of moisture on the grinding of natural calcite using a ball mill was investigated with the use of different methods such as X-ray diffraction and scanning electron microscopy [4]. The authors found that calcite powder with the addition of 1 and 10 mass % of water was easy to grind to submicron size, but more importantly, a much lower degree of contamination was achieved compared to dry milling. In [5], authors investigated the comminution of dolomite at several different moisture levels with laboratory high-pressure roll mills. The performed study allowed the authors to determine the impact of input material moisture on product characteristics, specific energy consumption, also on applied load, roll gap, and roll speed.

The influence of particle moisture on the separation process was addressed in [11], where the authors proposed a wetted wall cyclone, which allowed them to increase separation efficiency. The significantly expanded research in this area is presented in [12]. The authors analyze the influence of particle moisture on particle size distribution, overall efficiency and grade efficiency in an axial cyclone separator. The conclusion is that the grade efficiency improves with higher moisture of material but only for particles of diameter over 10 μm. For smaller diameters the contrary trend was observed. However, the study focuses exclusively on the separation of dust with a diameter of 0–50 μm with moisture content not exceeding 0.3%. In review [13], the authors note that air classifiers fed with ground minerals usually perform best when input moisture does not exceed 1.5–2%, or 1–1.5% for more sticky materials.

All the aforementioned research was carried out using very different types of equipment, different loose materials and process parameters. Generalising the obtained results to other cases may lead to wrong conclusions, which justifies research based on specific equipment, products and process parameters.

As was mentioned before, grinding is an essential process for multiple industries. Thus, they continuously search for better comminution methods: cheaper, more energy-efficient, faster, quieter, allowing to shape particle features and so on. One of recent inventions in this field is electromagnetic mill [14]. It is a three-phase inductor of rotating electromagnetic field which moves small ferromagnetic elements (grinding media) inside a cylindrical working chamber. The device is capable of fast, fine and ultrafine grinding in continuous-flow or batch manner, in gas or liquid environments [15,16,17]. Target raw materials are mostly non-organical granular substances [14].

**Figure 1:**
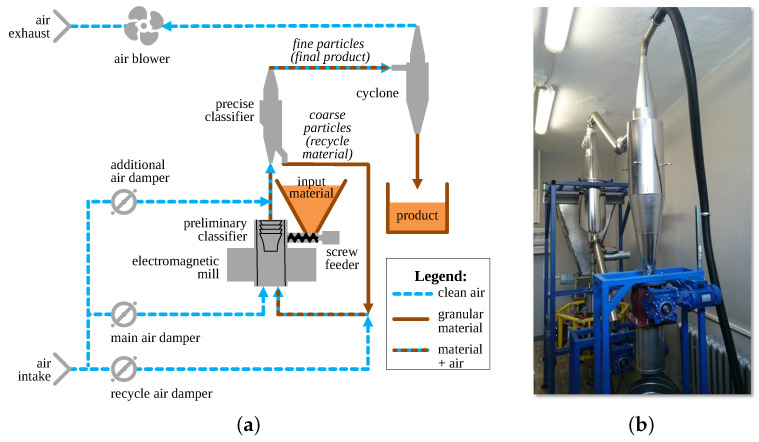
Installation for dry grinding with electromagnetic mill: (**a**) diagram, (**b**) photo–with cyclone in the foreground and precise classifier in the background. Credits: (**a**)–by authors, (**b**)–by Szymon Ogonowski.

An innovative complete grinding system employing the electromagnetic mill is a dry grinding circuit with recycle of too coarse material, underpressure transport system and layered automatic control system, as described in [16,18] and shown in Figure 1.

Input material and grinding media enter the working chamber of the mill through a screw feeder. After grinding, the particles are lifted by the air towards the classifiers (separators). The precise one is an inertial-impingement classifier. It divides the airborne particles into two streams: recycle (coarse particles) and final product (fine particles). The latter are separated from the transport air in the cyclone and then collected in a tank. The whole installation is equipped with numerous direct and indirect sensors measuring flows, temperatures, pressures, air humidities, material moistures, consumed electrical power, fill level of the working chamber, particle size distribution and other quantities (see e.g., [16,18]). These measurements are used by a hierarchical control system built of programmable logic controllers (PLCs) and the Supervisory Control And Data Acquisition (SCADA) system [16,18]. The layered structure and complexity of the control system creates a need for various models on which the control algorithms may rely.

The methods of online moisture measurement for input material and product streams in grinding system with the electromagnetic mill were already studied by the authors of present publication in [9,19]. However, material moisture needs to be monitored also in other parts of the installation—inside the grinding chamber, throughout the pneumatic transport pipeline, etc. In these locations, direct measurements are difficult or impossible to make because of harsh environment. So, moisture in these parts of the installation needs to be modelled (estimated), not measured. The first attempt to solve this modelling problem for the classification subsystem of the grinding circuit was reported by the authors in [20]. It proposed a general model of material moisture changes in the installation. The model was divided into: (1) moisture model of the electromagnetic mill with humidification system for the recycled material, and (2) moisture model of the material classification and separation path. The experimental results obtained in the study were approximated by using polynomials of 4th order. Such a model is not well suited for practical implementation in installation control algorithms due to its nonmonotonicity. In addition, the choice of such a model was based on limited statistical analyses. Herein, the authors intend to make an in-depth analysis of this issue and try a different approach to modelling.

## 2. Materials and Methods

### 2.1. Moisture Model of the Installation

Analyses of operation of the dry grinding installation with electromagnetic mill (Figure 1) led to a model describing the changes of water content (gaseous and liquid) in individual elements of the system [20]. Due to complex structure of such a model, the installation was divided into two parts for which separate moisture–humidity models were created:

- Model of the electromagnetic mill subsystem includes moisture/humidity changes in the mill itself with the integrated preliminary classifier and an additional moistening system.
- Model of the classification subsystem includes the precise classifier and the separating cyclone.

**Figure 2:**
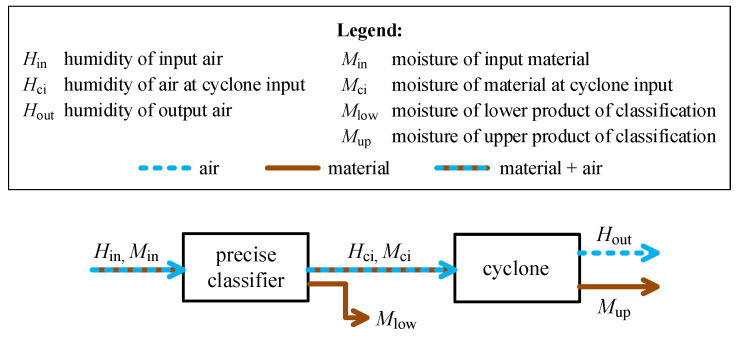
Moisture model (block diagram) of the classification subsystem.

The latter model, shown in Figure 2, is the basis of the experimental setup used in this research.

### 2.2. Installation

**Figure 3:**
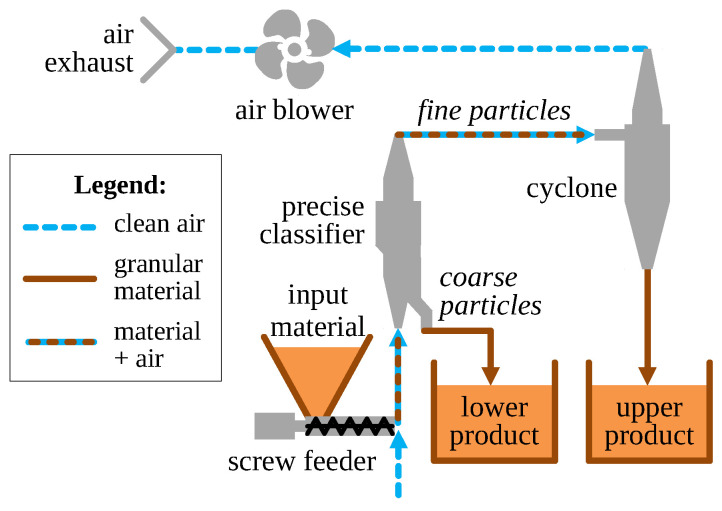
Experimental setup involving classification subsystem of the grinding circuit.

The experimental setup is shown in Figure 3. It consisted mainly of the classification subsystem of the grinding installation from Figure 1, i.e., precise classifier, cyclone and blower with the appropriate transport pipes and measurement sensors (not shown for figure clarity).

Moistened granular material (detailed in Section 2.3) was supplied from a screw feeder directly to the air stream entering the classifier. This material emulated the particles leaving the mill (working chamber + preliminary classifier). The throughput of the screw feeder was controlled by changing its rotational speed using a variable frequency drive (VFD). Both fine and coarse product of separation were collected in tanks for measurements. (Coarse particles were not recycled to the mill, as it was in the normal operation of the grinding circuit.)

### 2.3. Granular Material

**Figure 4:**
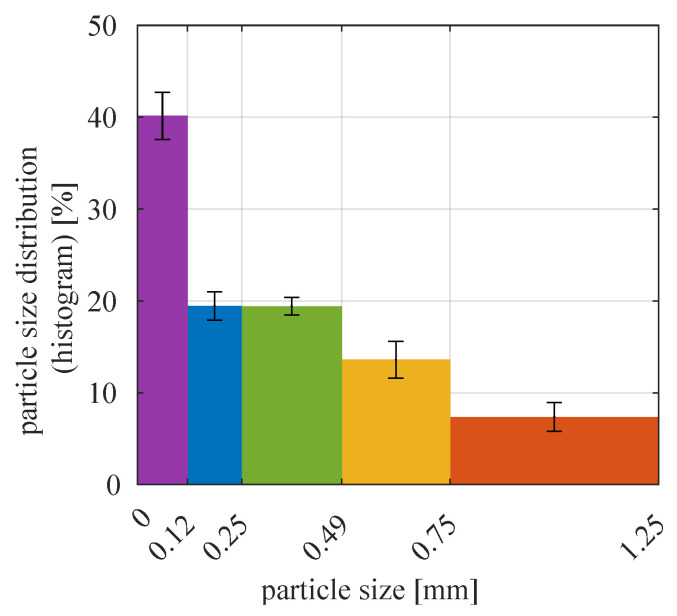
Histogram of particle size distribution for input material. Color bar heights indicate mean values for all experiments and error bars extend to ±1× standard deviation.

The material used during experiments was carbonate copper ore, one of the target substances of the described grinding and classification circuit. Particles of the input material were sized 0–1.25 mm; their particle size distribution is detailed in Figure 4.

The majority of fine particles and lesser content of coarse ones corresponded well to the fact that in the complete grinding circuit, the input material for the classifier was the output of the mill. Small standard deviations indicated that particle size distribution of the input material was homogeneous for all experiments (particle sizes were not affected by mixing of the material nor by transport through the experimental installation).

The above size distribution was obtained using manual sieve analysis. Currently researched machine vision techniques for assessment of particle size distribution [21,22] may radically shorten the duration of this measurement.

Generally, visual particle detection and classification can be performed based on geometric parameters such as area and diameter. Color and texture can also be useful, especially when considering the active surface of metal ore. This is the case in a method based on proper lighting and aperture control to extract metal surfaces on ore particles, developed in [21,22]. Firstly, paper [21] investigated machine-vision-based method of particle detection and classification in electromagnetic mill system for a wide range of particle sizes, shapes and positions in the prepared sample. The proposed method was developed for an on-line procedure with angle lighting and a simple image processing algorithm. Numerous particle shape features were investigated with the final selection of Heywood, compactness and aspect ratio factors. Then, in [22] the authors improved the method of material quality checking based on a cascade of two-dimensional Fast Fourier Transform (2DFFT) and Gray Level Co-occurrence Matrix (GLCM), combined Seeded Region Growing (SRG) with boundaries information from edge detection, and proposed a modified Niblack homogeneity algorithm. Moreover, the development of the proposed method will be concentrated on detecting the active surface of copper ore, and on determining the relationship between material moisture and granularity. In particular, the problem of considering material moisture requires extensive research, because material moisture, especially in the case of small particle sizes, may cause the formation of particle aggregates as a result of their mutual sticking. This directly affects the quality of particle classification, which depends on the number and shape of grains. The problem of the formation of aggregates is specific not only to the grinding process, but also to the sedimentation process [23,24].

### 2.4. Experiment Plan

Using a mixing paddle mounted on a drill, the material was intensely mixed with an appropriate amount of distilled water to achieve desired moisture. Tested moisture levels ranged from about 0.5% to about 5% (relative moisture) in different experiments. Higher values were also tested, but then the material was too wet to properly move inside the installation—it was sticking to the inner surfaces of pipes and other elements. Thus, the mentioned moisture range is the full range that may be used in the classification circuit.

The moistened material was supplied as the classifier input in an approximately constant air stream: ca. 2600–3000 L/min. That gives air velocities of ca. 3.4–3.9 m/s through the classifier (main pipe of 127 mm diameter) and ca. 7.6–8.8 m/s through the most of the pipeline (86 mm diameter). Air humidity (ca. 20–28%) and temperature (ca. 18–23 °C) at air intake were also approximately constant. Input material was supplied in two test series: with the screw feeder running at 50% and 100% of its nominal throughput. This corresponded to VFD frequencies of 25 and 50 Hz. The mass of supplied material was about 1.5 kg for the half-throughput experiments and about 3 kg for the full-throughput experiments. That resulted in about 3–4 min of material flow through the installation during each test run. Thus, material mass flows were ca. 15–25 kg/h at half throughput and ca. 30–45 kg/h at full throughput.

Both output streams from the classification process—i.e., lower product (coarse particles) and upper product (fine particles)—were collected in separate tanks for further examination. The content of each tank was carefully mixed to ensure proper homogeneity of the material. Then, samples were collected to measure moisture content and particle size distribution. Afterwards, all the material was mixed together and re-moistened for use in the next experiment.

Each moisture measurement (for the input material, lower and upper product) was performed on three material samples. They weighed about 8 g each and were taken from different regions of the collection tank. The measurements were carried out by means of thermogravimetric method (precise weighing of wet and dried material), using moisture analyzer RADWAG MA 110.R. For the details of the measurement process, see [19]. For the full set of collected moisture data, see supplementary materials, Tables S4–S9.

Particle size distribution of lower and upper classification product was assessed using manual sieve analysis with sieve holes of size 0.75 mm, 0.49 mm, 0.25 mm, 0.12 mm. Material samples used at this stage weighted ca. 80–100 g. They were thoroughly dried in a laboratory oven before sieving. The remains on the sieves were weighed with METTLER TOLEDO ICS425k-6SM/DR/f compact scale. Measured values are listed in supplementary materials, Tables S2 (lower classification product) and Tables S3 (upper classification product). Moreover, element-wise sums of the data in these two tables lead to particle size distribution of the input material, which is presented in Figure 4.

## 3. Results

### 3.1. Influence of Input Material Moisture on Separation Process

Weights of granularity classes for upper and lower product, measured in each experiment, may be used to calculate the so-called partition curve of the classifier. It indicates how much of each class was directed to the lower product (or to the upper product, depending on the adopted definition) ([25], pp. 89–91). For experiment number *e* and for each *i*-th granularity class, the corresponding point on the partition curve (the degree of separation) $PC_{i,e}$ is defined by [1,26]:

(1) $$PC_{i,e}=\frac{m_{\mathrm{low},i,e}}{m_{\mathrm{low},i,e}+m_{\mathrm{up},i,e}}\cdot 100\%=\frac{m_{\mathrm{low},i,e}}{m_{\mathrm{in},i,e}}\cdot 100\%\;,$$

where $m_{str,i,e}$ is the mass of *i*-th granularity class in material stream str during *e*-th experiment, where str={low,up,in}={lowerproduct,upperproduct,inputmaterial}. These are the masses presented in supplementary materials, Tables S2 and S3. In the plots of partition curves, the abscissae for degrees of separation are usually the middles of particle size intervals for each class [1]. Please note that each point of the curve is calculated independently of others, i.e., the curve is not a cumulative distribution of any quantity, though very often it is monotonically increasing. In addition, due to such definition (1), degrees of separation may be compared among different experiments (even if particle size distribution of input material was varying).

**Figure 5:**
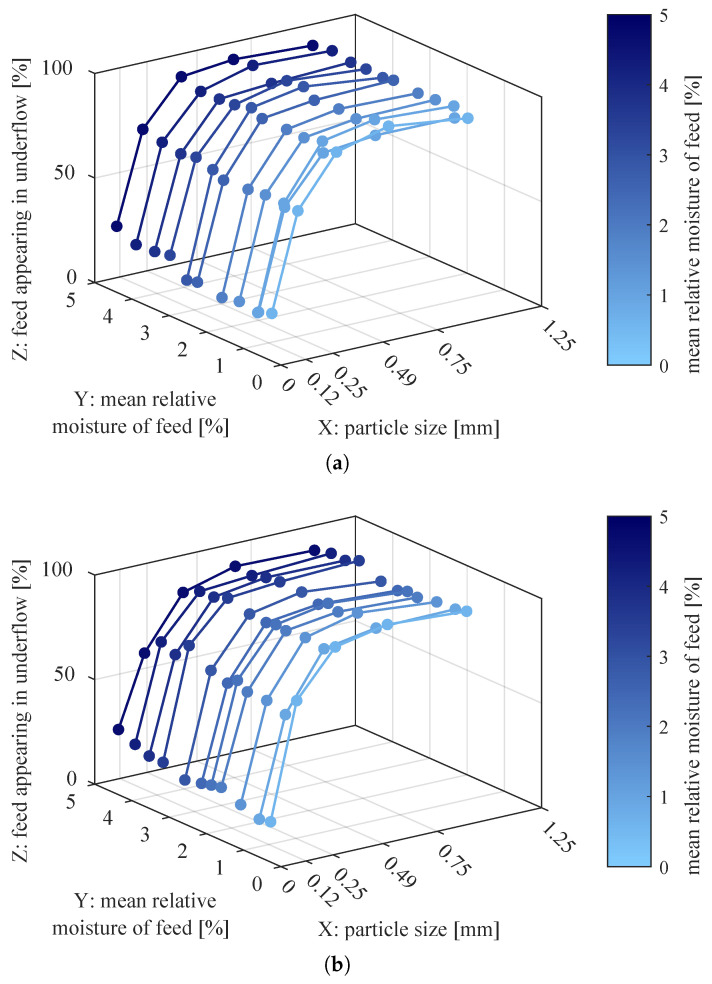
Partition curves for separator fed with material of varying moisture content. The material was supplied at: (**a**) 50%, (**b**) 100% of nominal throughput of the screw feeder.

To observe how changing moisture affects the partition curves, water content was added to the plots as the third dimension (Figure 5). The moisture abscissa for each curve is the average value of the three relative moistures measured for the input material in the particular experiment. In the following, the axes of these plots will be called: particle size–*X* axis; moisture content in the input material–*Y* axis; degree of separation (value of partition curve)–*Z* axis.

Each partition curve (seen as the XZ cross-section of the 3D plot) had the usual shape obtained for impingement-inertial classifier, i.e., it could be approximated by Weibull distribution [26] or scaled arctangent function [1]. This suggests that operating conditions of the separator were correct during the experiments and that sieve analysis was properly performed.

**Figure 6:**
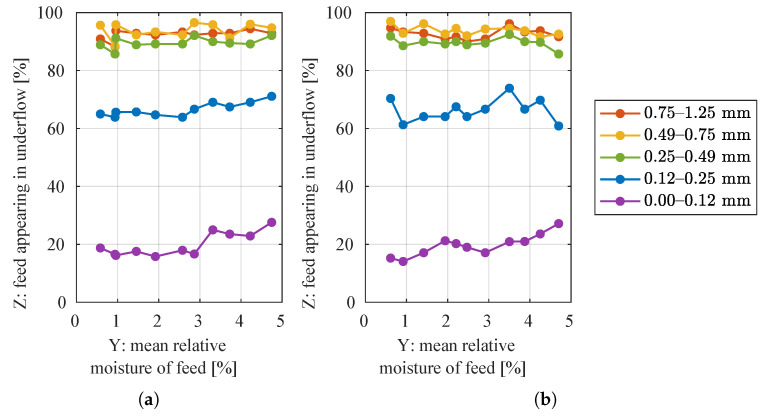
Degrees of separation from each experiment grouped by granularity class, in relation to input material moisture. The material was supplied to the separator at: (**a**) 50%, (**b**) 100% of the nominal throughput of the screw feeder.

On the other hand, the YZ cross-sections of the above 3D plots (Figure 5) show the relation between the input material moisture and the degree of separation for a selected granularity class, i.e., the relation between water content in successive experiments and $PC_{i,e}$ for fixed class number *i* and varying experiment number *e*. These relations are better visible in Figure 6.

The coarser particle classes seemed to have rather constant degree of separation; however, the finer particles tended to appear in lower product more often when there was more water in the material. The strength of the relationship between input moisture and degree of separation was evaluated with Pearson’s product–moment correlation coefficient $r_{P}$ [27] and Spearman’s rank correlation coefficient $r_{S}$ [28] (see Appendix A). The former coefficient measures linear correlation between variables and the latter—any monotonic correlation. The calculated coefficients were also tested for statistical significance with Student’s *t*-test (see Appendix A). Level of significance α was selected as 0.05 and there were (number_of_data_points−2)=11−2=9 degrees of freedom. Thus, *t*-test critical value $t_{1-\alpha }=t_{95\%}=2.26$. The calculated coefficients and results of *t*-test for the measured data are displayed in Table 1.

The monotonic correlation for particles of size 0.25–0.49 mm at 50% throughput was statistically significant, but not very strong. For other cases of small particles, if the correlations were significant, they were also quite strong (coefficient values mostly around 0.8).

### 3.2. Influence of Separator and Cyclone on Moisture of Product Streams

**Figure 7:**
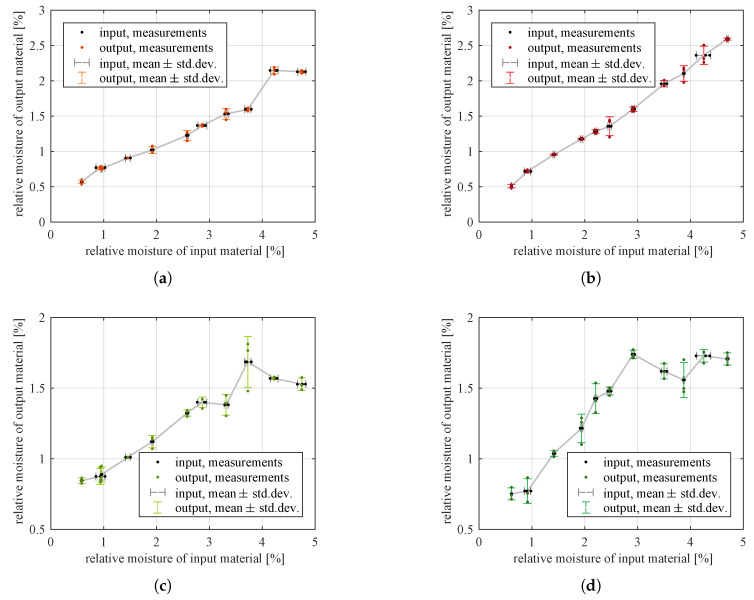
Measured moisture of both classification products related to moisture of input material, separately for different throughputs of the screw feeder: (**a**) lower product, 50% of nominal throughput; (**b**) lower product, 100% of nominal throughput; (**c**) upper product, 50% of nominal throughput; (**d**) upper product, 100% of nominal throughput. Points indicate three measurement attempts for each quantity in each experiment, error bars extend to ±1× sample standard deviation of the three measurements, cross-sections of horizontal and vertical error bars mark the averages of the three measurements.

Measured moistures of input and output material in each experiment are listed in supplementary materials, Tables S4–S9 and visualized in Figure 7.

The measured moistures of output materials—lower than moisture at the input—indicated that the particles were dried by the transport air during their travel through the transport pipes, the classifier and (in the case of upper product) the cyclone. To monitor and control the moisture of the material in the entire process, it was desirable to find a model for the relationship between moistures of input and output material. To make this model easily applicable in control algorithms running on PLCs, it should be simple and it may be approximate, rather than being very accurate but much complicated. Thus, a straight line model is a reasonable choice for a first attempt. The uncertainties (standard deviations) of measured moistures varied between data points and these uncertainties were present both in predictor variable (input material moisture) and in response variable (lower or upper product moisture). So, an ordinary least squares (LS) algorithm would not yield optimal estimates of model parameters; a weighted modification of the LS algorithm is needed to account for these errors [29] [Appendix 10D]. Among others, Cantrell [30] studied and compared several propositions from the literature and he found that the iterative method developed in [31] was accurate and convenient for use, and that it estimated also standard errors of parameters (not only parameter values). Hence, the method [31] was applied to the moisture data.

Details of the algorithm may be found in the original paper [31]. The implementation for this particular research was the following:

1. predictor variable was the average input material moisture from each experiment,
2. response variable was each single measurement of product moisture from each experiment,
3. the initial weights for values of predictor and response variables were set to reciprocals of sample variances (where each sample variance was calculated from three measurements made in each experiment),
4. the initial value of the slope of the line was estimated with ordinary least squares method.

Such initial values (points 3 and 4 above) were suggested in [31]. For each *i*-th data point, the initial weights $w_{x,i}$ and $w_{y,i}$ for predictor and response variables were combined by the algorithm into a single weight $W_{i}$. Then, these weights and the slope of the line were iteratively recomputed until the estimated slope converged (i.e., until the slopes estimated in subsequent iterations differed by less than the selected tolerance). For moisture data, this tolerance was selected as $10^{-10}$, which resulted in convergence after 5–7 iterations, depending on the data set (upper or lower product, 50% or 100% of feeder throughput).

The estimated model coefficients with descriptive statistical measures are given in Table 2. The weighted mean squared error (WMSE) shown in the table, used to compare the goodness of fit of different models to measured data, is defined as:

(2) $$\mathrm{WMSE}=\frac{\sum_{i=1}^{N}W_{i}\cdot {\left(y_{i}-{\hat{y}}_{i}\right)}^{2}}{\sum_{i=1}^{N}W_{i}}\;,$$

where: *N*—number of data points, $y_{i}$—real (measured) output variable (moisture of lower or upper product at 50% or 100% of nominal screw feeder throughput) at *i*-th data point, ${\hat{y}}_{i}$—model output at *i*-th data point, $W_{i}$—final weight assigned to *i*-th data point.

Goodness of fit was also indicated in the table using coefficient of determination $R^{2}$ and adjusted coefficient of determination $R_{\mathrm{adj}}^{2}$ (see Appendix B for details).

**Figure 8:**
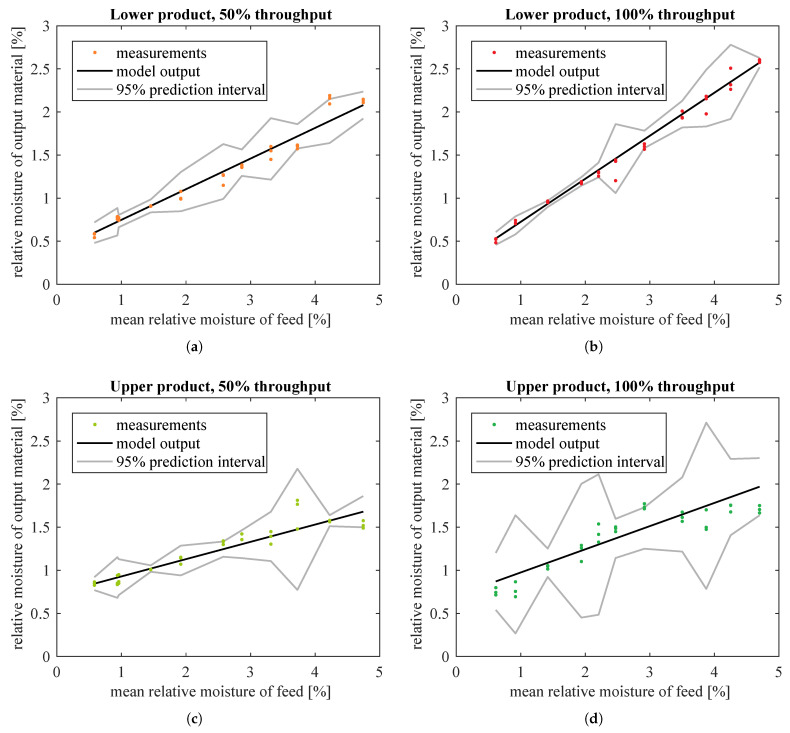
Straight line models fitted to measured moisture of classification products in relation to moisture of input material, separately for different products and different throughput of the screw feeder: (**a**) lower product, 50% of nominal throughput; (**b**) lower product, 100% of nominal throughput; (**c**) upper product, 50% of nominal throughput; (**d**) upper product, 100% of nominal throughput.

The fitted lines are plotted in Figure 8 together with their 95% prediction intervals. Prediction intervals are an estimation of range of values in which a single measurement falls with the given probability. They were calculated according to [32], as explained in Appendix C.

**Figure 9:**
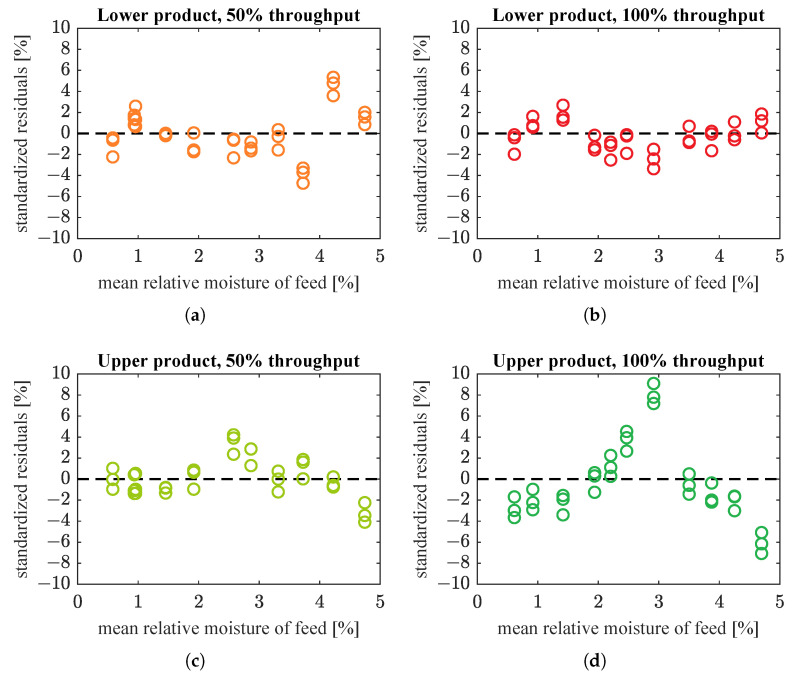
Residual plots for straight line models from Figure 8: (**a**) lower product, 50% of nominal throughput; (**b**) lower product, 100% of nominal throughput; (**c**) upper product, 50% of nominal throughput; (**d**) upper product, 100% of nominal throughput.

In addition, Figure 9 presents residual plots for these straight line models. In the plots, the residuals $e_{w,i}$ are standardized with the above-mentioned weights $W_{i}$ [33]:

(3) $$e_{w,i}=\sqrt{W_{i}}\cdot \left(y_{i}-{\hat{y}}_{i}\right)\;.$$

Residual plots still exhibited some patterns, so straight lines seemed to be too simple to model the relationships between predictor and response variables. However, for lower product, the prediction intervals were not very wide. Thus, these models were a reasonable approximation of the true dependencies, good enough for the intended use in upper-layer control algorithms. For upper product, the prediction intervals were much wider (in relation to the range of measured outputs). This indicated that a better model needed to be found.

The scatter plots of moisture data for upper product (Figure 7c,d) suggested fitting a (still simple) model of a saturated straight line, where the saturation of output occurred for higher values of predictor variable. The following algorithm was used to find such a model:

1. Select a value of input variable $x_{b}$ which should become the boundary between the sloping and horizontal lines.
2. Fit a line ${\hat{y}}_{i}=a_{1}x_{i}+b_{1}$ to all data points at $x_{i}\leq x_{b}$ using the already introduced algorithm [31].
3. Given the slope and intercept of this best-fit line, calculate model output at $x_{b}$: ${\hat{y}}_{b}=a_{1}x_{b}+b_{1}$. This value becomes the coefficient of the horizontal line: ${\hat{y}}_{i}=0\cdot x_{i}+b_{2}={\hat{y}}_{b}$ which models the output signal for all inputs $x_{i}>x_{b}$.
4. Calculate the weights for data points at $x_{i}>x_{b}$ in the same way as the algorithm [31] would do. Using them, together with the weights previously calculated for points at $x_{i}\leq x_{b}$, calculate WMSE (2) for the whole dataset.
5. Search for optimal $x_{b}$ that minimizes WMSE for the given dataset: change $x_{b}$ and repeat steps b–d until the optimum is reached.

For moisture data, the initial value of $x_{b}$ (step 1 above) was set to 3, based on scatter plots in Figure 7; and optimization (step 5 above) was done with Nelder–Mead direct search method, as implemented in MATLAB function fminsearch.

**Figure 10:**
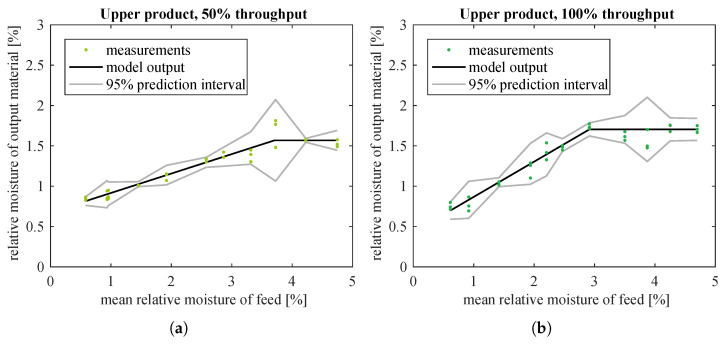
Straight lines with saturation fitted to measured moisture of upper classification product in relation to moisture of input material, separately for: (**a**) 50%, (**b**) 100% of nominal throughput of the screw feeder. Data for lower product are not plotted as they are identical to Figure 8a,b.

**Figure 11:**
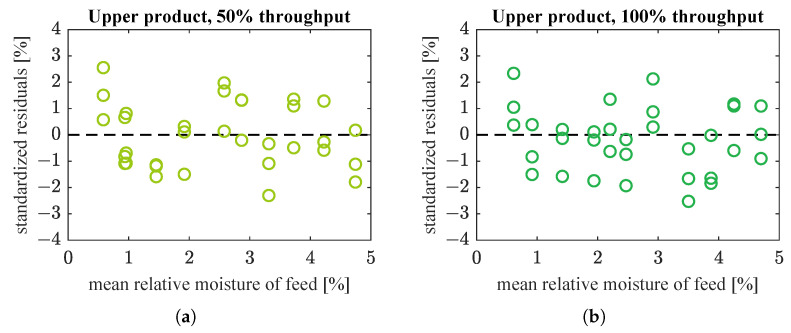
Residual plots for straight lines with saturation from Figure 10: (**a**) upper product, 50% of nominal throughput; (**b**) upper product, 100% of nominal throughput. Data for lower product are not plotted as they are identical to Figure 9a,b.

The estimated model coefficients and goodness-of-fit indices are given in Table 3. Fitted models and weighted residuals are plotted in Figure 10 and Figure 11, respectively. Model estimation results (Table 3) show that saturation was not used in the case of lower product datasets and the best fitted model is then a straight line only. This is the same as in Table 2 and Figure 8 and Figure 9. Hence, the plots for lower product were not drawn again.

Models with saturation were substantially better fitted to moisture of upper product than straight line models. WMSE was significantly reduced: from $1.7\times 10^{-3}$ to $5.6\times 10^{-4}$ (67% decrease) and from $2.3\times 10^{-2}$ to $2.5\times 10^{-3}$ (89% decrease), respectively for data corresponding to half and full nominal throughput of the screw feeder. Simple and adjusted $R^{2}$ for these data sets were also improved, especially for the upper product fed at full throughput. (Compare Table 2 and Table 3.) In addition, with the new model the prediction intervals were slightly narrower for data at 50% material throughput, and considerably smaller for data at 100% throughput (compare Figure 8c,d with Figure 10a,b). Residuals had smaller amplitudes and formed much more randomized patterns than previously, which was also desirable (compare Figure 9c,d with Figure 11a,b). The model seemed accurate enough for use in the upper control layers.

**Figure 12:**
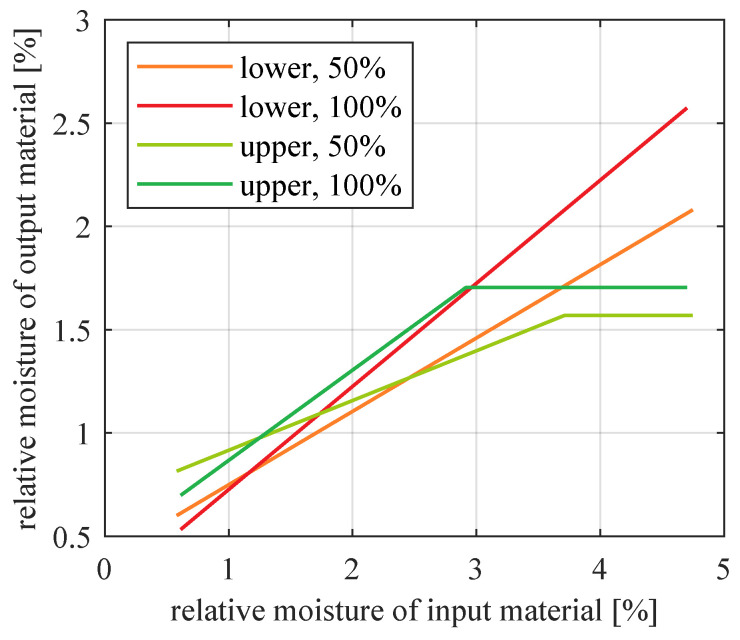
Comparison of models for lower and upper product of classification, for 50% and 100% nominal feeder throughput: straight line models for lower product and saturated straight line models for upper product.

**Figure 13:**
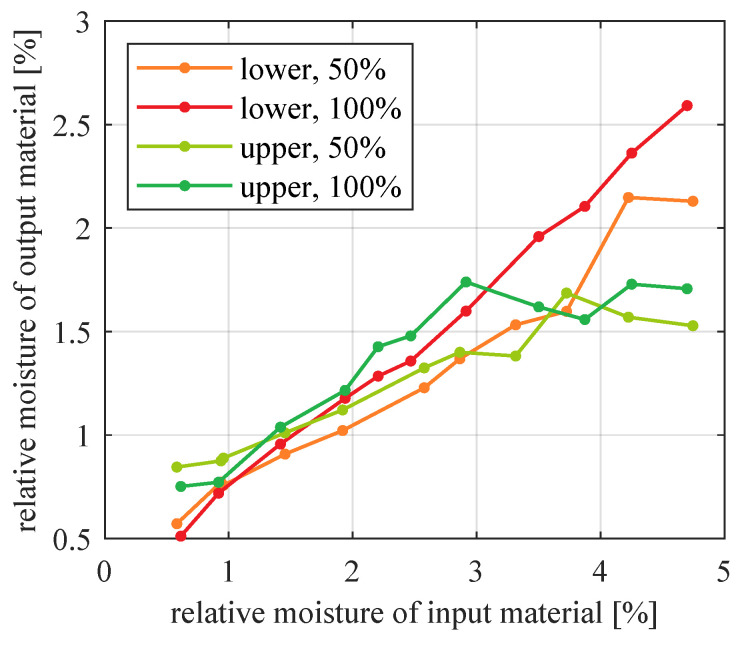
Comparison of measured moisture (average values) for lower and upper product of classification, for 50% and 100% nominal feeder throughput.

Outputs of the best models identified for each dataset are compared in Figure 12. For reference, all measured values are drawn in a single plot in Figure 13.

## 4. Discussion

### 4.1. Effect of Input Material Moisture on Separation Process

The data used for $r_{P}$ and $r_{S}$ calculation were measured with some uncertainty. Water content was measured using moisture analyzer with high-precision balance, so the uncertainty there is mostly associated with sampling of the measured material. Measurement uncertainties may be defined by standard uncertainty type A, i.e., sample standard deviations computed from the three measurements taken in each experiment. Uncertainties in calculated degrees of separation are much more difficult to assess because they have many sources:

- The scales accuracy (±1 g) contributes to two mass measurements $m_{\mathrm{low},i,e}$ and $m_{\mathrm{up},i,e}$ used in calculation of each separation degree (see (1)).
- The precision of sieve analysis is limited, especially for manual sieving. Each particle fraction retained at a sieve contains a slight amount of undersized particles, which should have fallen through the sieve. It is expected that bigger amount of material on the sieve causes more unwanted particles to remain, as with more material it is harder to reach the sieve screen for a single given particle. Thus, each sieve with coarser predecessor (i.e., each but the most coarse) is lacking a slight amount of input material; and each sieve with finer successor (i.e., each but the last bowl) is keeping a slight amount of excessive undersized particles. The lacking and excessive masses most probably do not cancel out completely. This phenomenon may be diminished by careful (prolonged and dynamic) sieving, but it can never be avoided.
- Moreover, sieve analysis was only done for samples of material. They were chosen carefully and are believed to be representative, but nevertheless they only sampled the whole amount of material.

Analyzing these sources of uncertainty, it is difficult to derive their cumulative description quantitatively and to use these values for formal assessment of uncertainty of correlation coefficients. Moreover, uncertainty evaluation is more complex for correlated quantities, as explained in [34]. However, the associations are quite strong (coefficient values are relatively high compared to significance thresholds). In addition, cumulative errors are random (not systematic, not proportional to the measured values). Taking these into account, it may be assumed with high probability that the correlations indeed exist.

This moisture–separation degree relationship may be due to extra weight added to the particles by the water (the operating principle of inertial-impingement classifier is actually to sort the particles by speed, which is related more to their weight, not strictly to their size). However, then all granularity classes (not only fine ones) should be affected. Perhaps water droplets adhere differently to smaller and bigger particles and thus affect only finer classes. Another possible explanation is that some of small moistened particles stick together (aggregate) into groups, which behave like bigger, heavier particles when subjected to classification.

The correlation revealed in these data is important for several reasons. If fine particles content in lower product grows, it means that cut size of the classifier decreases. (Cut size is a characteristic feature of a classifier and indicates particle size for which degree of separation (1) is 50% [1,26].) Particle size distribution of the final product (upper classification product) is modified (more fine particles), which may or may not be desired by the end user. In addition, the stream of recycle material increases, raising the load of the grinding chamber and lowering the overall throughput of the grinding circuit—all control subsystems have to react to this.

Please note that moisture content of granular material needs control for many purposes which have big impact on the whole grinding process (see Introduction). Thus, the conclusion from the above findings is not to manipulate moisture of material entering the classifier in order to change parameters of classification process. Instead, these observations allow for different control subsystems to prepare for the abovementioned changes in classifier behaviour (feed-forward control). Additionally, the identified relationships may suggest which moisture settings to choose if general requirements are somehow flexible (i.e., if desired moisture is a range of values and not a single value).

### 4.2. Effect of Separation Process on Moisture of Products

Moisture measurements indicate that bulk material gets dried by the transporting air when it undergoes separation process in particle classifier and cyclone. The resultant moisture content depends on the moisture of input material. For the coarse particles (lower product of classification), this relationship may be modelled by a straight line; for the fine particles (upper product of classification), a much more accurate model is a straight line with saturation at specific maximum value.

Most probably, the physical phenomena underlying the process are too complex to be modelled so simply—this is also suggested by residuals, which are not fully randomly distributed (Figure 9a,b and Figure 11). However, these models are accurate enough for use in upper and optimization layers of the control system, which determine proper operating conditions of the grinding installation. Simplicity of the models presented in this paper makes them effective and convenient to use for this purpose.

The positions of the calculated lines in relation to each other (Figure 12) reflect several phenomena which occur in the installation.

- Both lower and upper product are generally more moistened if more material is travelling through the pipes and tanks (i.e., if feeder throughput is higher). Of course this is because with more moistened particles, there remains more water which cannot be absorbed by the air.
- An exception is the range of very small input moistures (less than about 1.25%)–there, higher throughput results in lower output moisture. This may be related to surface moisture lost due to impact with other particles (the more particles, the more collisions).
- Moisture of upper product is saturated at about 1.6% relative moisture, but this phenomenon does not occur for lower product. One reason may be that the upper product goes through the cyclone and some additional pipes. This way, these particles have much longer contact with transport air. Their moisture has enough time to settle down, when exchange of water between material and air is finished. In contrast, coarse particles travel a very short path between the installation input and recycle material output. They do not have enough time to reach a similar steady state of water exchange. Another reason may be relatively small amount of water that fine particles manage to hold, compared to bigger particles. In practice, the observed saturation means that a moisturizer is necessary near the output of upper product if desired moisture is higher than ca. 1.6%. Please note that the specific value of this moisture saturation may differ for other experimental conditions, such as different material type, particle size, material and air mass flow, air humidity, temperature, etc.

## 5. Conclusions

This research evaluated the influence of input material moisture on particle separation process and the influence of separation process on moisture of lower and upper classification products. The proposed research has a strong practical meaning because it directly affects the control of the grinding process.

The authors developed an experimental setup based on the classification subsystem of a dry grinding installation. The setup included precise classifier (of inertial-impingement type) and separating cyclone. In addition, the usable range of material moistures has been determined in preliminary experiments, which was 0.5–5% relative moisture for the tested raw material (carbonate copper ore of 0–1.25 mm particles).

As a result of the research, several relevant dependencies have been distinguished that should be taken into account when controlling the grinding process. Firstly, statistical analysis showed that higher moisture levels increase fine particles content in lower product of classification for the analyzed type of classifier. In other words, high input material moisture reduces the cut size of the classifier. Attention was drawn to the impact that this has on the characteristics of the final product and on the course of the grinding process.

Secondly, this research enhanced the existing moisture model of the classification subsystem. The model describes drying of material which was observed during particle separation process. Straight line models were proposed for the relationships between moistures of input and lower classification product, and saturated straight line models—for the relations between moistures of input and upper classification product. The mathematical models proposed and verified herein are both simple and accurate enough to be used in control algorithms for the grinding installation.

## Figures and Tables

**Table 1:** Assessment of correlation between moisture level and degree of separation for each granularity class, separately for experiments with different material throughput of the screw feeder. $r_{P}$–Pearson’s correlation coefficient (A1), $r_{S}$–Spearman’s rank correlation coefficient (A2), $t_{r_{P}}$ or $t_{r_{S}}$–transformed correlation coefficient (A3), “sig.?”–is the result statistically significant at 95% confidence level?

**Particle Size**	**50% of Nominal Throughput**					
	$r_{P}$	$t_{r_{P}}$	$r_{P}$ **sig.?**	$r_{S}$	$t_{r_{S}}$	$r_{S}$ **sig.?**
0.75–1.25 mm	0.527	1.86	no	0.485	1.66	no
0.49–0.75 mm	0.246	0.761	no	0.251	0.778	no
0.25–0.49 mm	0.471	1.60	no	0.613	2.33	YES
0.12–0.25 mm	0.835	4.55	YES	0.795	3.93	YES
0–0.12 mm	0.812	4.18	YES	0.673	2.73	YES
**Particle Size**	**100% of Nominal Throughput**					
	**$r_{P}$**	$t_{r_{P}}$	$r_{P}$**sig**.?	$r_{S}$	$t_{r_{S}}$	$r_{S}$**sig.**?
0.75–1.25 mm	−0.0170	−0.0510	no	−0.0183	−0.0549	no
0.49–0.75 mm	−0.522	−1.83	no	−0.506	−1.76	no
0.25–0.49 mm	−0.335	−1.07	no	−0.165	−0.502	no
0.12–0.25 mm	0.111	0.335	no	0.0276	0.0828	no
0–0.12 mm	0.864	5.15	YES	0.802	4.03	YES

**Table 2:** Coefficients and statistical parameters of fitted straight lines between moisture of classification products and moisture of input material. *low*—lower product, *up*—upper product, 50% and 100%—percentage of nominal throughput of the screw feeder, SD–standard deviation, WMSE—weighted mean squared error (2), $R^{2}$—coefficient of determination (A5), $R_{\mathrm{adj}}^{2}$—adjusted coefficient of determination (A6).

	Data Set			
	*low*, 50%	*low*, 100%	*up*, 50%	*up*, 100%
**slope ${}^{a}$**	0.3553	0.4994	0.2005	0.2689
**SD of ${}^{a}$**	0.0040	0.0034	0.0032	0.0062
**intercept ${}^{b}$**	0.3935	0.2263	0.7285	0.706
**SD of ${}^{b}$**	0.0086	0.0085	0.0076	0.017
**WMSE**	0.0037	0.0012	0.0017	0.023
**$R^{2}$**	0.9810	0.9985	0.9965	0.9512
**$R_{\mathrm{adj}}^{2}$**	0.9804	0.9985	0.9964	0.9496

${}^{a}$ Number of model parameters used in Equation (A6): k=1, since saturation was not actually used. ${}^{b}$ Number of model parameters used in Equation (A6): k=2, since saturation was indeed used.

**Table 3:** Coefficients and statistical parameters of fitted saturated straight lines between moisture of classification products and moisture of input material. *low*—lower product, *up*—upper product, 50% and 100%—percentage of nominal throughput of the screw feeder, SD—standard deviation, *x*–model input, *y*—model output, WMSE—weighted mean squared error (2), $R^{2}$—coefficient of determination (A5), $R_{\mathrm{adj}}^{2}$—adjusted coefficient of determination (A6).

	Data Set			
	*low*, 50%	*low*, 100%	*up*, 50%	*up*, 100%
**slope ${}^{a}$**	0.3553	0.4994	0.2408	0.437
**SD of ${}^{a}$**	0.0040	0.0034	0.0072	0.011
**intercept ${}^{b}$**	0.3935	0.2263	0.675	0.430
**SD of ${}^{b}$**	0.0086	0.0085	0.012	0.022
**saturation for x≥…**	>5, so does not occur	>5, so does not occur	3.71	2.91
**saturation at y=…**	not applicable	not applicable	1.57	1.70
**WMSE**	0.0037	0.0012	0.00056	0.0025
**$R^{2}$**	0.9810	0.9985	0.9994	0.9944
**$R_{\mathrm{adj}}^{2}$**	0.9804 ${}^{a}$	0.9985 ${}^{a}$	0.9993 ${}^{b}$	0.9940 ${}^{b}$

${}^{a}$ Number of model parameters used in Equation (A6): k=1, since saturation was not actually used. ${}^{b}$ Number of model parameters used in Equation (A6): k=2, since saturation was indeed used.

## Data Availability

Not applicable.

## Supplementary Materials

Measurement data from the experiment are available online at https://www.mdpi.com/1424-8220/21/2/667/s1.

## Appendix A. Correlation Coefficients *r_P_* and *r_S_*

Pearson’s product-moment correlation coefficient $r_{P}$ [27] measures linear correlation between variables. Spearman’s rank correlation coefficient $r_{S}$ [28] indicates any monotonic correlation, since it calculates Pearson’s coefficient between ranks of values instead of the values themselves [35]. Both coefficients can take values from −1 to 1, where 1 signifies perfect positive correlation, −1 is perfect negative correlation, and 0 indicates no (linear or monotonic, respectively for $r_{P}$ and $r_{S}$) correlation. Fractional values mean there is weaker (not perfect) positive or negative correlation; the higher the absolute value of the coefficient, the stronger the association between variables.

If two quantities *x* and *y* form a sample of $\left(x_{i},y_{i}\right)$ points, i=1,2,…,N, then Pearson’s product-moment correlation coefficient is defined as:

(A1) $$r_{P}\left(x,y\right)=\frac{\sum_{i=1}^{N}\left(x_{i}-\bar{x}\right)\left(y_{i}-\bar{y}\right)}{\sqrt{\sum_{i=1}^{N}{\left(x_{i}-\bar{x}\right)}^{2}\cdot \sum_{i=1}^{N}{\left(y_{i}-\bar{y}\right)}^{2}}}=\frac{s_{xy}}{\sqrt{s_{xx}\cdot s_{yy}}}\;,$$

where $\bar{x}$ is the arithmetic mean of *x* values, $s_{xx}$ is the sample variance of *x* values, analogous definitions apply to $\bar{y}$ and $s_{yy}$, and $s_{xy}$ is the sample covariance of *x* and *y*.

For Spearman’s rank correlation coefficient, the following applies:

(A2) $$r_{S}\left(x,y\right)=r_{P}\left(r_{x},r_{y}\right)\;,$$

where $r_{x}$ are ranks assigned such that the samples $x_{i}$ are sorted in ascending order, then the minimum value of $x_{i}$ gets rank $r_{x,i}=1$, and the following $x_{i}$ get the next ranks up to $r_{x,i}=N$ corresponding to the maximum value of $x_{i}$. Similarly, ranks $r_{y}$ for all samples of *y* are assigned.

The resultant correlation coefficients may be tested for statistical significance with Student’s *t*-test. A transformed coefficient:

(A3) $$t_{r}=r\sqrt{\frac{N-2}{1-r^{2}}}\;,$$

where $r_{P}$ or $r_{S}$ is substituted for *r*, needs then to be compared with critical value $t_{1-\alpha }$ taken from Student’s *t*-distribution with N−2 degrees of freedom and a selected level of significance α. If $|t_{r}|>t_{1-\alpha }$, then the coefficient *r* is statistically significant at (1−α) confidence level [36].

## Appendix B. Coefficients of Determination $R^{2}$ and $R_{\mathrm{adj}}^{2}$

Goodness of fit of model output to measured data may be assessed with coefficient of determination $R^{2}$. For ordinary least squares—so, unweighted estimation—it is defined as [33]:

(A4) $$R_{\mathrm{unw}}^{2}=1-\frac{\sum_{i=1}^{N}{\left(y_{i}-{\hat{y}}_{i}\right)}^{2}}{\sum_{i=1}^{N}{\left(y_{i}-\bar{y}\right)}^{2}}\;;$$

where: *N*–number of data points, $y_{i}$–real (measured) output variable at *i*-th data point, ${\hat{y}}_{i}$–model output at *i*-th data point, $\bar{y}$–mean value of measured output.

When data points are weighted, as in the estimation method used in this research, this definition may be changed to [33]:

(A5) $$R_{w}^{2}=1-\frac{\sum_{i=1}^{N}{\left(y_{w,i}-{\hat{y}}_{w,i}\right)}^{2}}{\sum_{i=1}^{N}{\left(y_{w,i}-{\bar{y}}_{w}\right)}^{2}}=1-\frac{\sum_{i=1}^{N}W_{i}\cdot {\left(y_{i}-{\hat{y}}_{i}\right)}^{2}}{\sum_{i=1}^{N}{\left(\sqrt{W_{i}}\cdot y_{i}-{\bar{y}}_{w}\right)}^{2}}\;,$$

where: $W_{i}$ is the final weight assigned to *i*-th data point, $y_{w,i}=\sqrt{W_{i}}\cdot y_{i}$ and ${\hat{y}}_{w,i}=\sqrt{W_{i}}\cdot {\hat{y}}_{i}$ are weighted outputs of the plant and of its model, and ${\bar{y}}_{w}=\frac{1}{N}\sum_{i=1}^{N}y_{w,i}$ is the mean value of the transformed (weighted) measured output.

It is debatable whether formula (A4) or (A5) should be used (see e.g., [33]). The latter was adopted in the current research, as the authors believe that the unequal quality of measured data should be reflected in the computed indices.

The adjusted coefficient of determination $R_{\mathrm{adj}}^{2}$ [37] was also calculated. It accounts for the number of independent variables in the model and thus allows to compare different model structures:

(A6) $$R_{\mathrm{adj}}^{2}=1-\left(1-R^{2}\right)\cdot \frac{N-1}{N-1-k}\;,$$

where *k* denotes number of parameters in the model, excluding the free coefficient (so, for the straight line model, k=1). As $R^{2}$, formula (A4) or (A5) may be substituted. For consistence, again the latter was used in this research.

## Appendix C. Prediction Intervals for Straight Line Model When Measurement Data Have Errors on Both Axes

For the used modified least squares algorithm [31], the prediction intervals were calculated according to [32], with small adjustments (mainly in the notation, as in [32] the weights are used in reciprocal form and in [31], the weights are used directly).

The notation is: *N*–number of data points; $x_{i},\;y_{i},\;{\hat{y}}_{i}$–measured input, measured output and model output, respectively, at *i*-th data point (where input variable is the input material moisture and output variable is the classification product moisture); $s_{\left(\cdot \right)}^{2}$–estimated variance of the given signal; $W_{i}$–weight of *i*-th data point, as assigned by the identification algorithm [31]. In this research, variances of single measured values ($s_{x_{i}}^{2}$ and $s_{y_{i}}^{2}$) were estimated as sample variances from repetitive measurements, as three moisture measurements were made for each material sample.

The procedure for estimating prediction intervals is as follows:

- Variance $s_{e_{i}}^{2}$ of the modelling error $e_{i}$ at *i*-th data point may be estimated as:
  (A7) $$s_{e_{i}}^{2}=s^{2}\cdot \frac{1}{W_{i}}\;,$$
  where $s^{2}$ is a common factor between variances of the error at different data points and its unbiased estimate is a kind of weighted mean squared error [32]:
  (A8) $$s^{2}=\frac{\sum_{i=1}^{N}W_{i}\cdot {\left(y_{i}-{\hat{y}}_{i}\right)}^{2}}{N-2}\;.$$
  Note: Formula (A7) is changed compared to the literature method [32]: the cited paper uses $s^{2}$ alone and in (A7), multiplication by $\frac{1}{W_{i}}$ is added. This is because $s^{2}$ estimates the variance of weighted least squares residuals, i.e., of residuals scaled by weights (3); and to get back the individual variances of unscaled residuals, $s^{2}$ has to be divided back by the weights. This way, measurement points with bigger variance (i.e., with smaller weight) also have the corresponding errors with bigger variance.
- Variances of individual model outputs $s_{{\hat{y}}_{i}}^{2}$ may be estimated as [32]:
  (A9) $$s_{{\hat{y}}_{i}}^{2}=\left(1+X_{i}^{T}{\left(X^{T}WX\right)}^{-1}X_{i}+s_{x_{i}}^{2}a^{2}\right)\cdot s_{e_{i}}^{2}\;,$$
  where superscript T denotes matrix transpose; matrix $X=\left[\begin{array}{cc} 1 & x_{1}\\ \vdots & \vdots \\ 1 & x_{N} \end{array}\right]$; column vector $X_{i}={\left[1,x_{i}\right]}^{T}$; *a* is the estimated slope of the best-fit straight line.
  The term $s_{x_{i}}^{2}$, which in general case may vary irregularly between the data points, may cause the resultant prediction intervals to be not smooth [32]. This also occurs for moisture data analysed in this paper.
- For the selected probability (here, 1−α=0.95=95%), constant $t_{\alpha ,N-2}$ should be taken from Student’s *t*-distribution for significance level α and N−2 degrees of freedom. Then, the prediction interval $PI_{i}$ around ${\hat{y}}_{i}$ is [32]:
  (A10) $$PI_{i}={\hat{y}}_{i}\pm t_{\alpha ,N-2}\cdot s_{{\hat{y}}_{i}}\;.$$
