# Supplementary material for: Measurement-Based Modelling of Material Moisture and Particle Classification for Control of Copper Ore Dry Grinding Process
Source: Sensors (Basel). 2021 Jan 19;21(2):667. doi: 10.3390/s21020667 (PMC7833444; doi:10.3390/s21020667)
Supplement: Supplementary file 1 [file sensors-21-00667-s001.pdf]

Supplementary Materials: Measurement-Based Modelling of Material Moisture and Particle Classification for Control of Copper Ore Dry Grinding Process

Oliwia Krauze, Dariusz Buchczik and Sebastian Budzan

1. Screw Feeder Throughput During Experiments

Table S1. Throughput of the screw feeder (as percentage of nominal throughput) during experiments.

Throughput [%]	Experiment no.
50	2, 3, 5, 7, 9, 12, 14, 15, 17, 19, 22
100	1, 4, 6, 8, 10, 11, 13, 16, 18, 20, 21

2. Measurement Data: Material Weights

Table S2. Weights of subsequent granularity classes (particles retained on the sieves) measured after each experiment – lower classification product.

Experiment no.	Mass [g] of particles sized: *0–0.12 mm*	*0.12–0.25 mm*	*0.25–0.49 mm*	*0.49–0.75 mm*	*0.75–1.25 mm*	Total mass [g]
**1**	9	19	34	32	18	**112**
**2**	12.5	23	30	23	15	**103.5**
**3**	11	21	31	23	15	**101**
**4**	10	19	31	26	14	**100**
**5**	13	23	32	24	13	**105**
**6**	13	25	36	25	13	**112**
**7**	12	22	33	28	12	**107**
**8**	17	25	33	25	10	**110**
**9**	14	23	33	24	14	**108**
**10**	16	27	36	26	11	**116**
**11**	15	25	32	23	9	**104**
**12**	13	24	35	28	12	**112**
**13**	13	24	34	25	10	**106**
**14**	18	26	32	22	10	**108**
**15**	20	29	34	21	13	**117**
**16**	21	30	35	22	15	**123**
**17**	24	32	35	18	13	**122**
**18**	22	28	36	25	11	**122**
**19**	21	29	36	23	13	**122**
**20**	18	34	49	35	25	**161**
**21**	17	26	36	30	14	**123**
**22**	19	29	33	24	17	**122**

Table S3. Weights of subsequent granularity classes (particles retained on the sieves) measured after each experiment – upper classification product.

**Experiment no.**	**Mass [g] of particles sized:**					**Total mass [g]**
	0–0.12 mm	***0.12–0.25 mm***	***0.25–0.49 mm***	***0.49–0.75 mm***	***0.75–1.25 mm***	
**1**	50	8	3	1	1	**63**
**2**	63	13	5	3	2	**86**
**3**	57	11	3	1	1	**73**
**4**	61	12	4	2	1	**80**
**5**	61	12	4	2	1	**80**
**6**	63	14	4	1	1	**83**
**7**	64	12	4	2	1	**83**
**8**	63	14	4	2	1	**84**
**9**	64	13	4	2	1	**84**
**10**	63	13	4	1.5	1	**82.5**
**11**	64	14	4	2	1	**85**
**12**	65	12	3	1	1	**82**
**13**	63	12	4	1.5	1	**81.5**
**14**	78	14	4	1	1	**98**
**15**	65	14	4	2	1	**86**
**16**	68	13	4	2	1	**88**
**17**	63	13	3	1	1	**81**
**18**	59	18	6	2	1	**86**
**19**	63	13	4	1	1	**82**
**20**	68	12	4	2	1	**87**
**21**	64	13	4	2	1	**84**
**22**	64	13	4	1	1	**83**

Note: Weights of subsequent granularity classes for the *input* material used in *i*-th experiment were calculated as sums of corresponding weights for the upper and lower product.

3. Measurement Data: Material Moisture

3.1. Input Material

Table S4. Measurements needed to calculate the moisture of input material – screw feeder running at 50% of nominal throughput. Entries sorted by increasing average moisture.

Experiment no. (a)	Sample no. (b)	Mass [g] of wet sample (c)	Mass [g] of dried sample (d)	Mass [g] of water $(e) = (c)–(d)$	Moisture [%] $(f) = (e)/(c) \cdot 100\%$	Average moisture [%] $(g) = \frac{1}{3}\sum(f)$
	**1**	8.442	8.393	0.049	0.58	
**14**	**2**	8.606	8.555	0.051	0.59	0.58
	**3**	8.070	8.024	0.046	0.57	
	**1**	8.219	8.149	0.070	0.85	
**2**	**2**	8.307	8.222	0.085	1.02	0.94
	**3**	8.224	8.147	0.077	0.94	
	**1**	8.488	8.406	0.082	0.97	
**3**	**2**	8.525	8.444	0.081	0.95	0.96
	**3**	8.361	8.281	0.080	0.96	
	**1**	9.180	9.050	0.130	1.42	
**5**	**2**	8.805	8.678	0.127	1.44	1.45
	**3**	8.575	8.446	0.129	1.50	
	**1**	8.848	8.680	0.168	1.90	
**7**	**2**	8.109	7.952	0.157	1.94	1.92
	**3**	8.583	8.418	0.165	1.92	
	**1**	9.979	9.720	0.259	2.60	
**9**	**2**	7.026	6.846	0.180	2.56	2.58
	**3**	9.904	9.649	0.255	2.57	
	**1**	11.021	10.701	0.320	2.90	
**12**	**2**	6.670	6.475	0.195	2.92	2.87
	**3**	11.957	11.626	0.331	2.77	
	**1**	8.073	7.802	0.271	3.36	
**19**	**2**	10.938	10.577	0.361	3.30	3.32
	**3**	7.390	7.147	0.243	3.29	
	**1**	7.043	6.776	0.267	3.79	
**15**	**2**	8.046	7.750	0.296	3.68	3.73
	**3**	8.657	8.336	0.321	3.71	
	**1**	7.960	7.630	0.330	4.15	
**22**	**2**	8.694	8.322	0.372	4.28	4.23
	**3**	9.760	9.345	0.415	4.25	
	**1**	10.444	9.941	0.503	4.82	
**17**	**2**	7.250	6.905	0.345	4.76	4.75
	**3**	8.557	8.158	0.399	4.66	

Table S5. Measurements needed to calculate the moisture of input material – screw feeder running at 100% of nominal throughput. Entries sorted by increasing average moisture.

Experiment no. (a)	Sample no. (b)	Mass [g] of wet sample (c)	Mass [g] of dried sample (d)	Mass [g] of water $(e) = (c)–(d)$	Moisture [%] $(f) = (e)/(c) \cdot 100\%$	Average moisture [%] $(g) = \frac{1}{3}\sum(f)$
	**1**	8.377	8.326	0.051	0.61	
**1**	**2**	8.173	8.123	0.050	0.61	0.61
	**3**	8.243	8.192	0.051	0.62	
	**1**	8.361	8.279	0.082	0.98	
**4**	**2**	8.437	8.364	0.073	0.87	0.92
	**3**	8.376	8.300	0.076	0.91	
	**1**	8.483	8.364	0.119	1.40	
**6**	**2**	8.294	8.175	0.119	1.43	1.42
	**3**	8.345	8.227	0.118	1.41	
	**1**	6.532	6.407	0.125	1.91	
**8**	**2**	6.390	6.265	0.125	1.96	1.94
	**3**	7.495	7.349	0.146	1.95	
	**1**	8.383	8.196	0.187	2.23	
**10**	**2**	8.847	8.654	0.193	2.18	2.21
	**3**	8.576	8.387	0.189	2.20	
	**1**	6.120	5.967	0.153	2.50	
**11**	**2**	8.580	8.371	0.209	2.44	2.47
	**3**	8.215	8.012	0.203	2.47	
	**1**	9.677	9.392	0.285	2.95	
**13**	**2**	7.960	7.729	0.231	2.90	2.92
	**3**	9.697	9.416	0.281	2.90	
	**1**	6.417	6.189	0.228	3.55	
**20**	**2**	8.927	8.614	0.313	3.51	3.50
	**3**	9.287	8.967	0.320	3.45	
	**1**	9.680	9.304	0.376	3.88	
**21**	**2**	10.275	9.878	0.397	3.86	3.87
	**3**	9.503	9.135	0.368	3.87	
	**1**	9.785	9.358	0.427	4.36	
**16**	**2**	8.647	8.292	0.355	4.11	4.25
	**3**	8.306	7.950	0.356	4.29	
	**1**	7.855	7.487	0.368	4.68	
**18**	**2**	7.430	7.081	0.349	4.70	4.70
	**3**	8.101	7.719	0.382	4.72	

3.2. Lower Classification Product

Table S6. Measurements needed to calculate the moisture of lower product of classification – screw feeder running at 50% of nominal throughput. Entries ordered the same as in Table S4.

**Experiment no.** (a)	**Sample no.** (b)	**Mass [g] of wet sample** (c)	**Mass [g] of dried sample** (d)	**Mass [g] of water** $(e) = (c)–(d)$	**Moisture [%]** $(f) = (e)/(c) \cdot 100\%$	**Average moisture [%]** $(g) = \frac{1}{3}\sum(f)$
	**1**	8.490	8.440	0.050	0.59	
**14**	**2**	8.912	8.860	0.052	0.58	0.57
	**3**	8.115	8.071	0.044	0.54	
	**1**	8.396	8.330	0.066	0.79	
**2**	**2**	8.517	8.451	0.066	0.77	0.77
	**3**	8.482	8.418	0.064	0.75	
	**1**	8.334	8.272	0.062	0.74	
**3**	**2**	8.270	8.206	0.064	0.77	0.76
	**3**	8.484	8.420	0.064	0.75	
	**1**	8.381	8.305	0.076	0.91	
**5**	**2**	8.238	8.163	0.075	0.91	0.91
	**3**	8.490	8.413	0.077	0.91	
	**1**	8.488	8.404	0.084	0.99	
**7**	**2**	6.494	6.424	0.070	1.08	1.02
	**3**	6.716	6.649	0.067	1.00	
	**1**	6.404	6.323	0.081	1.26	
**9**	**2**	7.866	7.766	0.100	1.27	1.23
	**3**	8.354	8.258	0.096	1.15	
	**1**	8.477	8.362	0.115	1.36	
**12**	**2**	8.017	7.906	0.111	1.38	1.37
	**3**	8.425	8.310	0.115	1.37	
	**1**	7.486	7.370	0.116	1.55	
**19**	**2**	6.566	6.461	0.105	1.60	1.53
	**3**	6.623	6.527	0.096	1.45	
	**1**	5.673	5.582	0.091	1.60	
**15**	**2**	5.469	5.383	0.086	1.57	1.60
	**3**	5.692	5.600	0.092	1.62	
	**1**	9.404	9.198	0.206	2.19	
**22**	**2**	8.453	8.276	0.177	2.09	2.15
	**3**	6.898	6.749	0.149	2.16	
	**1**	8.251	8.075	0.176	2.13	
**17**	**2**	7.446	7.289	0.157	2.11	2.13
	**3**	7.729	7.563	0.166	2.15	

Table S7. Measurements needed to calculate the moisture of lower product of classification – screw feeder running at 100% of nominal throughput. Entries ordered the same as in Table S5.

Experiment no. (a)	Sample no. (b)	Mass [g] of wet sample (c)	Mass [g] of dried sample (d)	Mass [g] of water $(e) = (c)–(d)$	Moisture [%] $(f) = (e)/(c) \cdot 100\%$	Average moisture [%] $(g) = \frac{1}{3}\sum(f)$
**1**	**1**	8.425	8.381	0.044	0.52	0.51
	**2**	8.693	8.647	0.046	0.53	
	**3**	8.077	8.038	0.039	0.48	
**4**	**1**	8.495	8.432	0.063	0.74	0.72
	**2**	8.364	8.305	0.059	0.71	
	**3**	8.443	8.383	0.060	0.71	
**6**	**1**	8.376	8.295	0.081	0.97	0.96
	**2**	8.530	8.449	0.081	0.95	
	**3**	8.394	8.314	0.080	0.95	
**8**	**1**	8.728	8.626	0.102	1.17	1.18
	**2**	8.475	8.374	0.101	1.19	
	**3**	8.867	8.763	0.104	1.17	
**10**	**1**	8.263	8.156	0.107	1.29	1.28
	**2**	8.207	8.104	0.103	1.26	
	**3**	8.591	8.479	0.112	1.30	
**11**	**1**	8.458	8.336	0.122	1.44	1.36
	**2**	8.827	8.701	0.126	1.43	
	**3**	8.478	8.376	0.102	1.20	
**13**	**1**	7.974	7.849	0.125	1.57	1.60
	**2**	8.191	8.060	0.131	1.60	
	**3**	9.690	9.532	0.158	1.63	
**20**	**1**	9.748	9.552	0.196	2.01	1.96
	**2**	8.260	8.100	0.160	1.94	
	**3**	8.705	8.537	0.168	1.93	
**21**	**1**	9.484	9.277	0.207	2.18	2.10
	**2**	7.331	7.186	0.145	1.98	
	**3**	7.660	7.495	0.165	2.15	
**16**	**1**	8.493	8.280	0.213	2.51	2.36
	**2**	8.175	7.990	0.185	2.26	
	**3**	8.894	8.688	0.206	2.32	
**18**	**1**	9.206	8.966	0.240	2.61	2.59
	**2**	9.865	9.609	0.256	2.60	
	**3**	8.547	8.327	0.220	2.57	

3.3. Upper Classification Product

Table S8. Measurements needed to calculate the moisture of upper product of classification – screw feeder running at 50% of nominal throughput. Entries ordered the same as in Table S4.

**Experiment no.** (a)	**Sample no.** (b)	**Mass [g] of wet sample** (c)	**Mass [g] of dried sample** (d)	**Mass [g] of water** $(e) = (c)–(d)$	**Moisture [%]** $(f) = (e)/(c) \cdot 100\%$	**Average moisture [%]** $(g) = \frac{1}{3}\sum(f)$
	**1**	8.882	8.807	0.075	0.84	
**14**	**2**	9.018	8.940	0.078	0.86	0.85
	**3**	8.835	8.762	0.073	0.83	
	**1**	8.603	8.522	0.081	0.94	
**2**	**2**	7.194	7.134	0.060	0.83	0.88
	**3**	8.469	8.397	0.072	0.85	
	**1**	8.136	8.067	0.069	0.85	
**3**	**2**	8.173	8.102	0.071	0.87	0.89
	**3**	8.427	8.347	0.080	0.95	
	**1**	8.437	8.352	0.085	1.01	
**5**	**2**	8.395	8.310	0.085	1.01	1.01
	**3**	8.399	8.314	0.085	1.01	
	**1**	6.566	6.491	0.075	1.14	
**7**	**2**	6.070	6.005	0.065	1.07	1.12
	**3**	6.253	6.181	0.072	1.15	
	**1**	7.309	7.211	0.098	1.34	
**9**	**2**	5.473	5.400	0.073	1.33	1.32
	**3**	5.158	5.091	0.067	1.30	
	**1**	8.508	8.387	0.121	1.42	
**12**	**2**	8.370	8.251	0.119	1.42	1.40
	**3**	8.405	8.291	0.114	1.36	
	**1**	7.248	7.147	0.101	1.39	
**19**	**2**	7.363	7.267	0.096	1.30	1.38
	**3**	8.007	7.891	0.116	1.45	
	**1**	6.676	6.555	0.121	1.81	
**15**	**2**	5.435	5.339	0.096	1.77	1.69
	**3**	5.540	5.458	0.082	1.48	
	**1**	6.322	6.223	0.099	1.57	
**22**	**2**	7.740	7.619	0.121	1.56	1.57
	**3**	6.206	6.108	0.098	1.58	
	**1**	8.359	8.232	0.127	1.52	
**17**	**2**	6.980	6.870	0.110	1.58	1.53
	**3**	7.451	7.340	0.111	1.49	

Table S9. Measurements needed to calculate the moisture of upper product of classification – screw feeder running at 100% of nominal throughput. Entries ordered the same as in Table S5.

**Experiment no.** (a)	**Sample no.** (b)	**Mass [g] of wet sample** (c)	**Mass [g] of dried sample** (d)	**Mass [g] of water** $(e) = (c)–(d)$	**Moisture [%]** $(f) = (e)/(c) \cdot 100\%$	**Average moisture [%]** $(g) = \frac{1}{3}\sum(f)$
	**1**	8.393	8.326	0.067	0.80	
**1**	**2**	8.265	8.206	0.059	0.71	0.75
	**3**	8.210	8.149	0.061	0.74	
	**1**	8.475	8.411	0.064	0.76	
**4**	**2**	8.425	8.352	0.073	0.87	0.77
	**3**	8.501	8.442	0.059	0.69	
	**1**	8.378	8.293	0.085	1.01	
**6**	**2**	8.350	8.262	0.088	1.05	1.04
	**3**	8.505	8.416	0.089	1.05	
	**1**	8.587	8.479	0.108	1.26	
**8**	**2**	8.352	8.260	0.092	1.10	1.22
	**3**	8.301	8.194	0.107	1.29	
	**1**	8.472	8.352	0.120	1.42	
**10**	**2**	8.288	8.178	0.110	1.33	1.43
	**3**	8.327	8.199	0.128	1.54	
	**1**	8.314	8.189	0.125	1.50	
**11**	**2**	8.216	8.097	0.119	1.45	1.48
	**3**	8.682	8.553	0.129	1.49	
	**1**	8.856	8.699	0.157	1.77	
**13**	**2**	8.987	8.833	0.154	1.71	1.74
	**3**	9.121	8.963	0.158	1.73	
	**1**	6.685	6.573	0.112	1.68	
**20**	**2**	7.866	7.739	0.127	1.61	1.62
	**3**	10.332	10.170	0.162	1.57	
	**1**	6.985	6.882	0.103	1.47	
**21**	**2**	7.674	7.559	0.115	1.50	1.56
	**3**	10.636	10.455	0.181	1.70	
	**1**	7.814	7.677	0.137	1.75	
**16**	**2**	7.691	7.562	0.129	1.68	1.73
	**3**	7.970	7.830	0.140	1.76	
	**1**	7.710	7.575	0.135	1.75	
**18**	**2**	6.804	6.688	0.116	1.70	1.71
	**3**	6.605	6.495	0.110	1.67	
